# Determinants of Adherence and Satisfaction in Parkinson’s Disease Exercise Programs: A Comparison of 16-Months of Adapted Tango vs. Supervised Walking

**DOI:** 10.21203/rs.3.rs-6908014/v1

**Published:** 2025-06-20

**Authors:** Haneul Kim, Forouzan Rafiei, Meghan E. Kazanski, Amir Hossein Nekouei, Madeleine E. Hackney

**Affiliations:** Emory University; Emory University; Emory University; Kerman University of Medical Science; Emory University

**Keywords:** Parkinson, OFF State, Dyskinesia, FOG, Compliance, Satisfaction, Tango, Walk

## Abstract

Moderate aerobic activities are promising treatment modalities for mitigating motor and cognitive deficits associated with Parkinson’s disease (PD). This study aims to characterize the association of PD-specific characteristics with compliance and satisfaction to the moderate-aerobic activity. Thirty-six participants with PD engaged in either adapted Argentine tango (TANGO) (n=20) or supervised walking (WALK) (n=16) classes for 16 months. Participants attended between59 to 76 classes to be fully compliant. Metrics associated with PD characteristics, including frequency and duration of dyskinesia and OFF state, freezing of gait status (FOG), years since PD diagnosis, Hoehn-Yahr Stage and motor and cognitive function, were collected. Linear regression models were used to examine the association of the PD metrics with treatment compliance (number of completed classes) and self-reported program satisfaction composites. Program engagement analysis revealed a wide attendance distribution (range: 1–76 sessions; mean ± SD: 39.1 ± 26.0 sessions) over the 16-month intervention period. Overall participant satisfaction was favorable (mean ± SD: 4.0 ± 0.8 on a 5-point scale) across motor-cognitive, emotional, and psychosocial domains. Multivariate analysis identified significant negative correlations between dyskinesia metrics and program compliance, with both percentage of waking time with dyskinesia (β = −0.381, R^2^ = 0.145, p = 0.055) and total dyskinesia duration (β = −0.377, R^2^ = 0.142, p =0.058) emerging as key predictors. Program compliance demonstrated a positive association with participant satisfaction (β = 0.378, R^2^ = 0.143, p = 0.063). Hierarchical regression analysis of clinical predictors revealed cognitive function (MoCA scores) as the strongest predictor of participant satisfaction (β = 0.396, R^2^ = 0.157, p = 0.050), followed by non-motor daily living scores (UPDRS Part I; β = −0.343, R^2^ = 0.118, p = 0.093) and dyskinesia duration (β = −0.346, R^2^ = 0.120, p = 0.134). Secondary outcome measures including OFF-time duration, MDS-UPDRS total score, and disease duration showed minimal predictive value (all R^2^ < 0.10, p > 0.15). Notably, FOG demonstrated no significant impact on either program satisfaction (R^2^ = 0.022, p = 0.514) or attendance patterns (FOG-positive: 59.1 ± 21.4 vs. FOG-negative: 60.4 ± 18.9 sessions; t-test, p = 0.79). This study demonstrates that PD-related motor and cognitive characteristics significantly influence engagement in moderate-intensity aerobic interventions. While overall satisfaction was high, dyskinesia frequency and duration were associated with reduced compliance, indicating key barriers to participation. In contrast, cognitive functions emerged as the strongest predictor of satisfaction. However, FOG did not show any effect on program compliance or satisfaction. These findings highlight the need for tailored exercise programs that address motor limitations to enhance adherence and therapeutic outcomes.

## Introduction

PD is a long-term neurodegenerative disorder that profoundly hampers both motor and cognitive abilities, greatly diminishing quality of life (QOL) and general well-being^1,2^. PD ranks as the second most prevalent neurodegenerative condition worldwide, impacting around 2% of individuals over the age of 65, with its occurrence steadily rising due to the aging global population^3^. PD is caused by the degeneration of dopaminergic neurons in the basal ganglia and is characterized by motor symptoms, such as tremor, bradykinesia, muscle rigidity, and postural instability, and non-motor symptoms, such as depression, dysphagia, and urinary dysfunction^4^.

The manifestation of OFF episodes, defined as periods when medication effects wear off and motor symptoms worsen, during which pharmacological treatments lose their potency, symptom severity worsens, and the individual’s QOL is further diminished, represents a significant hurdle in the therapeutic management of PD^5^. Moreover, dyskinesias, involuntary motions triggered by prolonged use of levodopa, a defining motor-related adverse effect of extended levodopa therapy, can disrupt everyday tasks and pose challenges for planning effective treatment strategies^6^. Both OFF episodes and dyskinesias lead to unpredictable fluctuations throughout the day, greatly hindering individuals’ ability to carry out everyday tasks and adversely impacting levels of physical activity^7^. Medications such as levodopa can temporarily relieve symptoms, especially in the early stages of PD disease, but as the condition progresses, the treatment plan can result in drug-resistant complications, resulting in the return of symptoms of cognitive decline and even possibly medication-related problems maintaining balance. Therapeutic strategies must be broadened to incorporate supportive interventions and lifestyle adjustments^8^.

Regular physical activity has emerged as a promising supportive modality to address PD symptoms and potentially slow the progression of the disorder^8^. Moderate aerobic exercise has been repeatedly linked to improved motor skills, movement, and balance stability^8–10^. However, many current studies are constrained by brief intervention periods, typically lasting under six months and lack of extended follow-up assessments. As such, the long-term durability of these effects is largely unknown^11^. While observational evidence suggests that greater levels of regular physical activity may be associated with slower decline in motor and cognitive abilities^12^.

Over time, regular movement has increasingly been recognized as an indispensable component of thorough PD treatment. Numerous investigations have demonstrated that routine physical activity alleviates both motor-related and non-motor-related issues, enhances overall QOL, and could potentially slow the progression of the condition^13^. Despite the expanding body of evidence emphasizing these advantages, participation in fitness programs remains inadequate, encouraging researchers to examine the fundamental motivational and structural aspects influencing involvement.

Movement-related challenges, such as dyskinesia, Freezing of Gait and OFF-time, may pose significant barriers to maintaining consistent physical activity, as reduced treatment efficacy during these intervals often worsens mobility impairments and diminishes motivation^14,15^. Additionally, logistical obstacles, including transportation challenges, time limitations, and restricted access to exercise facilities, may further obstruct participation and decrease the perceived value of fitness-focused programs^13^.

In a comprehensive review, Schootemeijer et al. (2020) identified four categories of enablers and obstacles to exercise engagement within the framework of the International Classification of Functioning, Disability, and Health (ICF): bodily functions, tasks and involvement, individual traits, and environmental influences. Among the most notable physiological difficulties were exhaustion, muscle rigidity, involuntary shaking, indifference, and unease^13^. Ellis et al. (2013) conducted a study with 260 individuals diagnosed with PD, identifying three key deterrents to physical activity participation: low perceived advantages of exercise (OR = 3.93), insufficient time (OR = 3.36), and fear of losing balance (OR = 2.35), which were markedly more common among those who refrained from engaging in physical movement^16^.

Similarly, Hunter et al. (2019) conducted a qualitative systematic analysis and discovered that numerous individuals with PD worried that physical activity might exacerbate their symptoms or cause harm. However, such misconceptions were frequently addressed through targeted education and supportive group settings that emphasized the value of structured and safe exercise routines^17^.

Considering the substantial evidence emphasizing the beneficial impact of physical activity on improving motor abilities and physical performance in individuals with PD and recognizing that OFF periods frequently disrupt daily routines and hinder participation in structured exercise programs^14,15^. This research explores how condition-specific factors, particularly dyskinesia and OFF episodes, impact commitment to and satisfaction with moderate-intensity cardiovascular programs. Throughout a 16-month intervention, participants engaged in either a partner-supported dance-based aerobics session (TANGO) or a structured walking regimen (WALK). The findings from this assessment may contribute to the development of more inclusive, personalized recovery strategies that efficiently tackle both condition-specific restrictions and practical challenges, improving patient well-being and overall life quality within this expanding medical community.

## Methods

### Ethical Approval and Trial Registration

This study was approved by the Institutional Review Board of Emory University and the Research and Development Committee of the Atlanta Veterans Affairs Medical Center (VAMC). The trial was registered at ClinicalTrials.gov (NCT04122690), with full protocol details previously published^18^. As of drafting this report, this study has not been completed for all enrolled patients. Thus, this report represents a secondary analysis of a subset of the participants’ available data. A future primary report will detail the results, including all data.

### Participants and Recruitment

Participants were recruited through the Atlanta VAMC Movement Disorders Clinic using the VA Informatics and Computing Infrastructure (VINCI) database to identify eligible individuals within the Atlanta VA Health Care System. Additional participants were enrolled from the Emory University Movement Disorders Clinic, as well as through local PD support groups, educational events, and community outreach programs. At enrollment, participants underwent baseline assessments including general health screening, fall risk evaluation, demographic data (age, education), functional independence (activities of daily living), and global cognitive status using the MoCA.

### Inclusion and exclusion criteria

To be included, potential participants needed to present with a diagnosis of idiopathic (PD, which was confirmed by a board-certified neurologist specializing in movement disorders, according to established diagnostic criteria^19^. To participate, they must be over 40 years of age and belong to Hoehn and Yahr stages I to III, which represents the severity of PD. The key requirement is an OFF period with a score of 1 or higher on section 4.3 of the Unified PD Rating Scale (UPDRS-IV). This score represents the period during which the drug’s effectiveness decreases^18^.

The diagnosis of PD was confirmed using the ICD-10 code “G20” and further supported by clinical signs, such as unilateral onset and the presence of at least three key symptoms: rigidity, bradykinesia, tremor, or postural instability. A documented favorable response to antiparkinsonian treatments was also necessary to validate the diagnosis^20^.

Individuals with a Montreal Cognitive Assessment (MoCA) score below 18, indicating moderate to severe cognitive dysfunction, were excluded^21^.

### Informed Consent and Participant Flow

**Figure 1:**
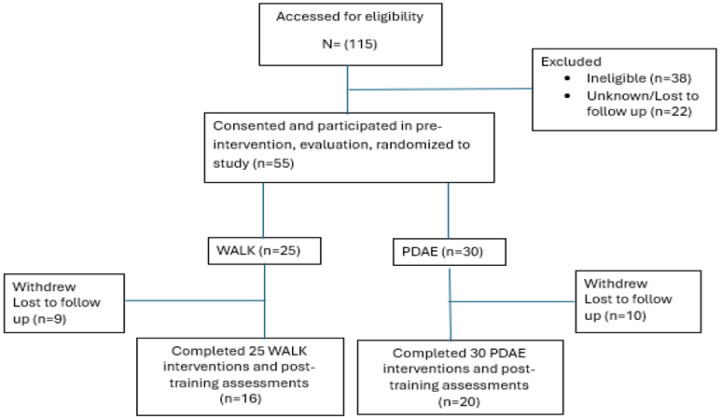
Consort Diagram at the time of Data Analysis for this Report

All participants provided written informed consent prior to study enrollment. Participant flow through the ongoing study is illustrated in Figure 1 (CONSORT diagram).

### Randomization and Intervention Assignment

36 participants who met all inclusion and exclusion criteria were enrolled and randomly assigned to one of two intervention groups: the TANGO Group (n = 20), which participated in partnered dance-aerobic exercise (PDAE) sessions, or the WALK Group (n = 16), who participated in a supervised, structured walking program. Participants engaged in their assigned exercise interventions over a 16-month period. Both interventions were delivered over a 16-month period and consisted of two distinct phases. Participants attended bi-weekly sessions for the initial 3-month Training Phase, for a total of 24 lessons, each lasting 90 minutes, resulting in approximately 180 minutes of moderate aerobic activity per week. For the remaining 13 months, during a Maintenance Phase, participants came less often and engaged in weekly sessions. During the Maintenance target of all participants were to participate in at least three sessions per month, amounting to 39 classes over the course of 13 months, with each meeting lasting 90 minutes. These classes included a 35-minute preparation and practice segment, 45 minutes dedicated to the core activity, and a concluding 10-minute cooldown phase. In total, the participants were to attend at least 63 sessions over the 16 months, but if they attended approximately 4 weekly sessions during Maintenance months, they would attend as many as 76 sessions.

### ADAPTANGO group

The PDAE intervention was based on an adapted Argentine tango program that has been previously shown to improve motor function, balance, gait, mobility^22^, endurance, and quality of life in individuals with PD^23^. Participants with PD were paired with non-PD partners such as trained caregivers, university students, or friends. Partners rotated every 15 minutes, and group sizes were capped at six pairs to prioritize safety and individualized attention. Sessions centered on exploring movement objectives through physical interaction, examining the connection between motion and rhythm, introducing fresh dance techniques, and integrating mastered and creative components. The instructional approach generally had minimal focus on memorization of fixed routines. Instead, each class introduced new steps to foster motor skill development and sustained engagement.

### WALK group

The WALK group received an equivalent duration, frequency, level of intensity, degree of oversight, and access to the same facility and trainers as the PDAE group. Each session consisted of a 25-minute preparatory phase, 45 minutes of walking, and a 10-minute cooldown, with integrated pauses for transitions. Walking exercises were mainly conducted outside on level ground, with extended indoor hallways used during unfavorable weather conditions. The WALK initiative took place in a group format, supervised by skilled researchers and volunteers to maintain safety and foster social engagement, aligning with the communal atmosphere of the PDAE program.

### Disease Severity and OFF-Time

At each evaluation time point, disease severity and motor fluctuations were assessed by a Movement Disorder Society (MDS)-certified rater blinded to participant allocation, using the MDS-Unified PD Disease Rating Scale (MDS-UPDRS I–IV)^24^. The MDS-UPDRS is divided into four sections: Part I examines non-motor elements of daily living, Part II focuses on motor-related experiences in everyday activities, Part III evaluates motor performance through a physical assessment, and Part IV concentrates on motor complications such as dyskinesia and OFF episodes. The principal evaluation tool utilized in this research is the MDS-UPDRS Part IV, with a specific focus on item 3 (duration of OFF episodes) and item 4 (effects of fluctuations on functional performance). Each item is scored on a range from 0 to 4, with higher values representing greater levels of disability.

To complement clinician-rated assessments, a three-day OFF-state diary^25^ was used monthly. Participants were instructed to categorize their motor state (OFF, ON, ON with disruptive dyskinesia, or sleep) and record every 30 minutes for 3 consecutive days. To measure OFF time, we determined the ratio of waking hours spent in the OFF and ON states accompanied by disruptive movement disorders. This self-reported approach reduces bias and accurately captures symptom fluctuations throughout the day. However, challenges such as adherence and memory limitations persist^26,27^. Integrating the MDS-UPDRS Part IV with the diary is intended to provide a comprehensive, peer-reviewed assessment of the intensity and complexity of OFF time.

### Cognitive Function

Cognitive status was assessed using the Montreal Cognitive Assessment (MoCA), a widely validated screening tool for detecting mild cognitive impairment in individuals with PD disease.

### Motor Function

Motor symptoms, including dyskinesia and FOG, were assessed using the full MDS-UPDRS I–IV. Dyskinesia burden was evaluated both as a percentage of waking hours and total daily duration, informed by both clinician scoring and diary data. FOG severity was measured using the Freezing of Gait Questionnaire^28^, a six-item instrument scored on a 24-point scale, with higher scores reflecting more frequent or disabling episodes. The disease stage was classified via the Hoehn and Yahr scale, which categorizes PD severity from stage 1 (mild symptoms) to stage 5 (severe symptoms requiring a wheelchair or resulting in being bedridden). These reliable assessments allowed for the measurement of both motor and non-motor symptom load pertinent to engagement in the intervention.

### Gait and Falls Risk

The risk of falling was assessed through the Gait and Falls Questionnaire, a 16-item tool evaluated on a 64-point scale (each item assigned a score from 0 to 4), with higher values reflecting an increased likelihood of falls. This assessment provided further understanding of mobility challenges linked to the progression of the disease.

### Participant Satisfaction

Upon concluding the intervention, a specially developed satisfaction questionnaire was distributed. Responses were scored using a 5-point Likert scale, assessing participants’ perceived motor-cognitive improvements, emotional gains, and psychosocial advantages derived from their involvement.

### Participant Satisfaction and Psychosocial Outcomes

At the conclusion of the intervention phase, participants engaged with a satisfaction questionnaire devised to evaluate subjective effects of program compliance. This instrument explored multiple dimensions, including perceived advancements in motor function and cognitive improvements, emotional resilience, and psychosocial benefits. Responses were gathered via a 5-tier Likert scale (1 = strongly agree to 5 = strongly disagree). This instrument was developed to evaluate self-reported outcomes that extend beyond clinician-led assessments or performance-based metrics. The resulting data provided valuable insights into the program’s acceptability, feasibility, and perceived significance from the participants’ perspective.

### Barriers to Participation and Program Logistics

To determine possible obstacles to program adherence, participants were surveyed about logistical issues, including transportation difficulties, scheduling challenges, and accessibility concerns. Responses were examined qualitatively to evaluate their impact on compliance. Physical activity intensity was tracked using wearable heart rate monitors, with the session “dose” calculated based on the total duration spent in class. Intervention fidelity was upheld through weekly instructor reports and monthly in-person reviews conducted by the study team. Additionally, all program staff received standardized training on PD-specific safety protocols, including posture support, balance tracking, and fall prevention, via a structured two-hour experiential workshop.

### Statistical Analysis

All analyses were conducted in RStudio 4.4.1. Descriptive statistics summarized baseline and outcome variables. Between-group comparisons for continuous variables were performed using independent t-tests, and categorical variables were assessed via chi-square or Fisher’s exact tests, as appropriate.

Associations between clinical variables (e.g., OFF-time, dyskinesia) and outcomes (compliance, satisfaction) were analyzed using Pearson correlation coefficients. For exploratory analyses, we identified associations that approached statistical significance (e.g., proportion and duration of dyskinesia). In addition, we utilized linear mixed-effects models to assess change over time, incorporating fixed effects for time, group, and group × time interactions, and a random intercept to allow participants to account for within-person variability. We provided the coefficient of determination (R^2^) for each model to measure explanatory power. Statistical significance was defined as 0.05, and marginal results (e.g., p = 0.055) were recognized as having potential clinical significance.

## Results

Descriptive demographic and clinical characteristics are summarized in Tables 1 and 2. There were no statistically significant differences between TANGO and WALK groups in age, sex, race, disease duration, Hoehn and Yahr stage, or PD-related motor complications, including OFF-time and dyskinesia at baseline (P>0.05). Compliance and satisfaction scores were also comparable at baseline. These findings confirm that randomization produced equivalent groups, allowing for an unbiased evaluation of intervention effects.

**Figure 2:**
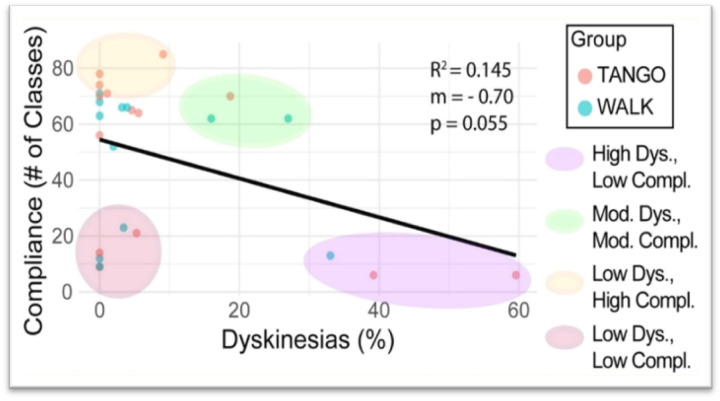
Associations Between Dyskinesia Percentage and Program Adherence

The association between dyskinesia percentage and program compliance (measured by the total count of attended exercise sessions throughout the intervention period) is shown in Figure 2. Individuals with greater levels of dyskinesia were inclined to attend fewer sessions. Participants with minimal dyskinesia demonstrated a wide range of compliance rates, whereas participants with elevated dyskinesia percentages tended to attend fewer classes (Figure 2).

### The strength of association between multiple PD-related clinical variables and program compliance was shown in Fig3.

The relationship between dyskinesia percentage, dyskinesia duration, years since diagnosis, Hoehn-Yahr stage, and OFF-time duration with class attendance is depicted. Among these, dyskinesia percentage and dyskinesia duration showed the strongest negative correlations with compliance (R^2^ = 0.145 and 0.142, respectively), both with near-significant p-values (p = 0.055 and p = 0.058, respectively). In contrast, other variables demonstrated weaker associations. Years since diagnosis and OFF-time duration had low R^2^ values and p-values exceeding 0.3. Hoehn-Yahr stage, a commonly used clinical staging measure, showed an almost flat trendline, with an R^2^ of 0.004 and a non-significant p-value (p = 0.731), indicating negligible association with attendance. Dyskinesia-related variables were more predictive of participation than overall disease duration or stage.

#### Linear Regression Analysis of Parkinson’s Disease-Related Clinical Characteristics as Predictors of Program Satisfaction

**Figure 6:**
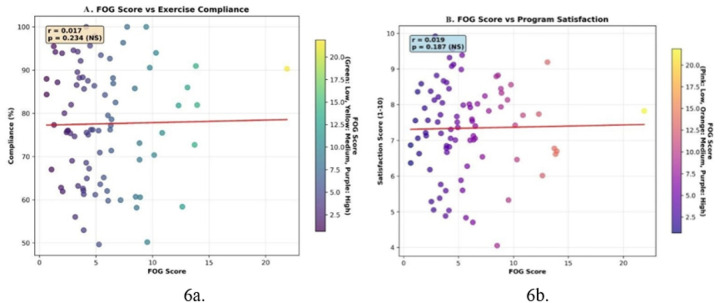
a. FOG score vs. Exercise Compliance, b. FOG score vs. program satisfaction

An examination of the linear regression analysis, aimed at predicting program satisfaction (the outcome variable) using Parkinson’s disease-related clinical characteristics as predictors, revealed that among the analyzed predictors, the Montreal Cognitive Assessment (MoCA) score, a measure of cognitive ability, exhibited the strongest association with participant satisfaction. This variable accounted for 15.7% of the variance in satisfaction scores (R^2^=0.157), with the relationship being at a borderline significance level (p=0.050). Furthermore, FOG did not appear to have a significant impact on participant experience and satisfaction (p > 0.05). Also, our data show that FOG severity does not significantly affect exercise compliance or satisfaction (Figure 6).

#### Linear Regression Analysis of Parkinson’s Disease-Related Clinical Characteristics as Predictors of Program Compliance

In the analysis predicting program compliance, where the number of sessions attended served as the dependent variable and clinical characteristics were the predictors, the dyskinesia index (both rate and duration) most consistently predicted decreased participation. The coefficient of determination (R2) for dyskinesia rate was 0.145 (p=0.055), and for dyskinesia duration, it was 0.142 (p=0.058). While these p-values narrowly miss the conventional 0.05 significance threshold, their proximity suggests a notable trend, indicating that these aspects of dyskinesia explain approximately 14.5% and 14.2% of the variance in session attendance, respectively, and are associated with lower program participation (Figure 6).

## Discussion

This study examined the influence of Parkinson’s disease-related clinical factors, including dyskinesia and OFF episodes, on participants’ engagement and satisfaction with two 16-month moderate-intensity cardiovascular programs: TANGO and WALK.

Our findings revealed that participants were highly content with the activity program, reporting positive experiences throughout its duration. Compliance was also notably high, with most participants consistently attending sessions and adhering to the prescribed exercises. The findings provide important insights into exercise participation among individuals with PD, showing that (1) higher levels of movement impairment, such as dyskinesias, can reduce participation in exercise and (2) sustained engagement in exercise participation could potentially impact the progression or severity of movement impairments over time. In a 16-month comparison of tango and walking exercise, we found that factors had a greater impact on outcomes than exercise methods. In particular, higher Montreal Cognitive Assessment (MoCA) scores were most strongly associated with program participation, with longer test times and participation being more welcome (R^2^=0.142–0.145) for those with movement impairment (R^2^=0.157). Different dimensions of one’s own exercise participation are likely to have different impacts.

### Dyskinesia and Program Compliance

We found a negative correlation between dyskinesia severity and participation in the TANGO and WALK programs. The disruptive effects of dyskinesia may operate through multiple pathways: physical interference with movement patterns, increased fall risk during complex exercises, and potential embarrassment in social exercise settings. This observation is consistent with research indicating that involuntary movements have a particularly strong effect on tasks demanding fine motor precision^29,30^. Participants with more pronounced dyskinesia showed lower attendance rates, whereas those with milder symptoms demonstrated a range of participation patterns, from regular involvement to occasional attendance. This emphasizes the subtle influence of symptom severity on adherence. These results reinforce prior research indicating that non-motor factors, such as logistical challenges, accessibility, motivation, and significantly influence adherence to exercise programs for individuals with PD^14^. Addressing these barriers is critical to improving participation rates, as low expectations of outcomes, fear of falling, and time constraints remain persistent challenges^14^.

In line with the findings of Politis et al. (2010), dyskinesia emerged as a significant obstacle to participation, closely linked to a decline in quality of life and functional abilities^5^. These results provide additional evidence reinforcing earlier studies, such as those by Politis et al. (2010), which associate dyskinesia with diminished quality of life and reduced functional capabilities. The erratic nature of dyskinesia can prompt patients to experience involuntary movements during exercise, potentially disrupting their workout routines. Interestingly, our results are consistent with those of Mantri et al. (2021), who identified challenges in sustaining exercise routines, rather than lack of access, as the primary obstacle to participation. The extended duration of our intervention could account for the differences in outcomes observed between the studies^31^. This difference may be due to differences in the duration of the intervention or the design of the program between the long-term intervention period (16 months) in this study and the short-term study that focused on partner dance rather than general exercise.

Although dyskinesia may be a barrier to participation, FOG does not significantly affect exercise participation. In addition, our results show that severity of FOG cannot predict adherence or satisfaction with moderate aerobic exercise. Contrary to hypotheses, Hoehn and Yahr stage and disease duration did not significantly correlate with participation levels. This suggests that movement complications, particularly dyskinesias, may play a more important role than overall disease severity in predicting adherence. These results are consistent with Hackney and Earhart (2010a), who found that tailored interventions, such as flexible scheduling, significantly improved participation in patients with severe symptoms^22^. From a mechanistic perspective, movement fluctuations, particularly dyskinesias, may interfere with participation by interfering with movement planning and increasing fall risk. Our findings support the “dual-task interference” hypothesis, in which movement complications exacerbate cognitive-motor trade-offs, particularly in complex activities such as dancing.

While dyskinesia presents an intrinsic barrier, extrinsic motivators such as peer support and group camaraderie may help offset its negative emotional impact, fostering a more inclusive exercise environment. For instance, Schootemeijer et al. (2020) showed that group camaraderie enhanced adherence by alleviating the distress associated with motor fluctuations, indicating that social support can help cushion the emotional challenges of dyskinesia.^13^.

### Dyskinesia and Other Disease-Related Factors

The study revealed that the severity of dyskinesia was a strong predictor of both program participation and its overall effectiveness. On the other hand, the same PD-related factors as time since symptom onset, Hohen-Yahr stage, and OFF status were found to be associated with participation and a weaker level of participation. This refutes the initial assumption indicating that dyskinesia significantly limits physical activity participation and contributes to reduced quality of life in individuals with PD. These results are consistent with the original findings that exercise participation is an important limiting factor for exercise participation in people with PD. Politis (2010) demonstrated that exercise involvement, including dyskinesia^5^, significantly influenced the use of PD emergency services and quality of life. This refutes the initial assumption indicating that dyskinesia significantly limits physical activity participation and contributes to reduced quality of life in individuals with PD.

These results are consistent with a study by Hackney and Earhart that investigated the impact of motor symptoms, including dyskinesia, on exercise participation in people with PD^22^. Their study found that people with more severe motor symptoms, such as dyskinesia, had significant difficulties in adhering to exercise routines. Hackney and Earhart highlighted the need for tailored interventions, including flexible scheduling, tailored support, and alternative exercise-based therapies, to effectively engage people with PD show frequently experience long-term dyskinesia.

### Participant Satisfaction

Our findings of the study revealed that participants were highly content with the activity program, noting self-assessed progress in physical, mental, and emotional areas. These outcomes align with previous research conducted by Rafferty, in 2017^32^. Everyone participant (100%) expressed that they enjoyed the activities, and 84% indicated their intent to continue participating. However, the effect felt was different for each person. While the majority (64%) responded that they had experienced an improvement in their walking ability, 24% responded that they had not noticed any such change, showing that improvements related to walking were not seen equally in everyone. Conversely, most participants saw positive changes in their mental clarity and emotional well-being, with 84% reporting increased engagement with their thoughts. This suggests that although the program has been generally well received, specific benefits vary depending on disease progression and individual differences in exercise capacity.

Moreover, Carmo (2024) highlighted the personalized effectiveness of exercise programs, especially for patients with PD^33^. According to Carmo, exercise programs that take into account an individual’s exercise symptoms, fitness level, and preferences can increase participation and satisfaction. Exercise routines tailored to meet specific needs, such as strength, endurance or mobility, are said to result in participants feeling greater benefits, which encourages continued participation^33^. Our results are consistent with Carmo’s study, where adjustments reflecting individual preferences, such as a focus on posture and endurance training, improved program performance, particularly among those with more severe symptoms or at different stages of PD^33^.

The findings from our study reveal exceptionally positive feedback from participants about the moderate-aerobic exercise program. All participants (100%) strongly agreed that the program provided numerous advantages, such as enhanced balance, gait, mood, coordination, strength, stamina, and increased physical as well as mental activity. The elevated satisfaction levels highlight the program’s efficacy in delivering substantial motor and cognitive improvements for individuals with PD. Additionally, our data demonstrate that participants with greater adherence reported higher satisfaction, emphasizing the critical role of regular involvement in achieving better outcomes^18^. Aligning with previous research, our results indicate that individuals with infrequent dyskinesia were more likely to comply with the program, highlighting the necessity for personalized support to encourage participation among those experiencing more severe symptoms^10^.

### Perspectives of Participants with moderate Compliance and moderate Dyskinesia on Aerobic Activity Program

Participants with moderate dyskinesia shared overwhelmingly positive feedback, with all respondents (100%) strongly agreeing that they enjoyed the program. These positive feelings extended across a range of domains, including improved balance, walking ability, mood, endurance, and mental performance. However, opinions regarding improvements in coordination and strength were slightly more mixed, with 67% strongly agreeing. These results are consistent with those of Cavanaugh (2015), who demonstrated that exercise programs for people with PD generally yield beneficial outcomes, but that the degree of benefit may vary depending on the severity of the condition^7^.

For those managing moderate dyskinesia, this program seemed to provide meaningful support for both physical and mental well-being, resulting in noticeable enhancements to their overall quality of life. The strong engagement and high satisfaction rates highlight just how valuable such initiatives are, underscoring the need to not only sustain them but also to expand their reach, ensuring more individuals can benefit from these kinds of opportunities. Tailoring exercise interventions to individual needs is crucial, as it allows for adjustments that accommodate varying motor functions and disease stages, ensuring that the program remains effective and engaging for participants with diverse symptoms and progression levels^7^.

From a psychological and social standpoint, the interpersonal connections fostered through partnered dance could ease feelings of discontent in individuals with moderate dyskinesia. This might clarify the elevated satisfaction reported by tango participants, even amidst motor difficulties. The social aspect of tango likely played a pivotal role in nurturing emotional wellness, bolstering participants’ drive to remain engaged in the program despite the physical constraints imposed by their condition.

### Perspectives of Participants with Low Compliance and Low Dyskinesia on Aerobic Activity Program

This section outlines feedback from participants with limited adherence and mild dyskinesia regarding their experiences in the moderate-aerobic activities program. Although all participants (100%) unanimously agreed that they enjoyed the program, opinions on specific improvements were more divided. Despite these differences, 67% strongly affirmed, and 33% expressed neutrality, that their level of physical activity had increased. These observations align with Nasreddine (2005), who proposed that cognitive shifts in PD progress gradually and are often challenging to identify without formal assessment. The neutral feedback on mental engagement in this study likely reflects the slow progression of cognitive alterations, which may be difficult to assess solely through self-reported methods^21^. Furthermore, the limited engagement and diverse perspectives on program benefits highlight the critical need for individualized support strategies. Such approaches should aim to address specific challenges encountered by participants, tailoring the program to accommodate each individual’s distinct motor and cognitive capabilities to enhance its overall effectiveness.

### Compliance and Satisfaction in Tango and Walk

Our research highlighted clear distinctions between the tango dance and walking interventions, showcasing differences in participant involvement and adherence. The tango group, characterized by its interactive and social aspects, appeared to foster stronger mental engagement and emotional fulfillment, especially among individuals with moderate dyskinesia. The intricate nature of the dance, coupled with the social connections inherent in the activity, likely enhanced enjoyment and cognitive stimulation. This observation aligns with previous studies emphasizing the advantages of comprehensive exercise programs for people with PD^34^. In contrast, the walking group offered a straightforward and predictable exercise alternative, appealing to those who preferred a less strenuous activity. While walking ensured steady attendance, participants with milder dyskinesia displayed varied progress, particularly in areas like balance and stamina. These differences underscore the critical need for a variety of therapeutic options tailored to individual preferences and requirements, taking into account the motor function difficulties associated with PD.

The conclusions of our study align closely with those of Hackney and Bennett (2014)^23^, who highlighted the significance of diversity within fitness programs for individuals with PD. Their work suggested that incorporating both social and physical components into therapeutic approaches enhances patient commitment and involvement. Moreover, McKay (2016) identified that intricate activities like dance can boost cognitive engagement, potentially explaining the heightened satisfaction and mental stimulation reported by participants in our tango cohort^34^. The interactive aspect of tango likely played a crucial role, delivering not only physical exercise but also opportunities for social connection, which may have positively impacted mood and overall participation, as evidenced by our findings. Studies have demonstrated that the social element of physical activity, particularly in group environments, is a significant driver of sustained involvement over the long term. Interaction with peers, the sense of community, and the formation of social bonds are essential for maintaining engagement in physical activities^35^. These insights underline the importance of weaving these components into exercise interventions for individuals with PD, promoting lasting commitment and participation.

However, our research demonstrates some distinctions compared to previous findings. While numerous studies have underscored the advantages of dynamic activities such as dance (e.g., tango) for individuals with PD, our outcomes suggest that walking, despite its straightforward nature still serves as a valuable intervention for certain participants. The consistent and predictable aspects of walking appeared to resonate with individuals who favored less mentally taxing exercises. This diverges from the common assumption that more intricate activities, like dance, would universally excel in encouraging engagement and enhancing results. One plausible explanation for this variation lies in the individual differences in participants’ preferences and physical capacities. Whereas dance may stimulate greater cognitive involvement, walking provides a more approachable and steady option, particularly for those who may find complex exercises daunting or those managing more severe motor impairments. These varying responses highlight the significance of tailored rehabilitation strategies, blending both intricate and straightforward activities to accommodate the unique conditions and preferences of each individual.

### Cognitive and Non-Motor Influences on Compliance and Satisfaction

Further examination of clinical indicators influencing participant satisfaction identified cognitive abilities (assessed via MoCA scores), duration of dyskinesia, and non-motor components of daily living (measured by MDS-UPDRS I) as the most notable factors associated with satisfaction, with R^2^ values ranging from 0.118 to 0.157 and p-values below 0.10. The findings suggest that, in addition to motor irregularities, cognitive difficulties and non-motor issues play a crucial role in shaping participants’ views of program advantages. Although dyskinesia severity consistently emerged as the strongest predictor of adherence and satisfaction, the influence of cognitive capacity and the burden of non-motor symptoms highlight the intricate and multidimensional nature of exercise outcomes in PD. This underscores the need to design exercise interventions that not only address physical limitations but also provide mental engagement and flexibility to accommodate the varied symptoms and progression levels of participants.

### Satisfaction and Compliance Correlation

This study investigated the association between satisfaction and adherence within a moderate aerobic exercise program. The results are consistent with previous research by Hauser et al. (2004), which found that satisfaction was closely related to the patient’s perceived intervention effectiveness throughout the day, making it an important measure for assessing outcomes^25^. The evaluation uncovered a favorable association between contentment and adherence, suggesting that higher satisfaction could be linked to increased participation. However, this relationship did not reach statistical significance, implying that other influences, such as external challenges or individual drive, may affect commitment.

Furthermore, Hunter (2019) highlighted that negative experiences or observing patients with severe symptoms may lead to a reluctance to continue exercise^17^. This finding stresses the importance of creating a supportive environment and fostering positive interactions between patients and instructors. Programs like Rock Steady Boxing^36^ and Tai Chi^37^, have demonstrated that engaging, group-based exercise formats are more likely to improve participation rates and long-term adherence. These programs have also been associated with improvements in quality-of-life indicators, including reductions in fatigue, fear, and depression.

Beyond clinical factors, earlier studies have pinpointed substantial logistical obstacles affecting participation. Challenges like difficulties with transportation, conflicts in session scheduling, and restricted access to sports facilities posed significant hardships, particularly for individuals experiencing more severe symptoms^13^. Additionally, Ellis (2013) highlighted deterrents such as a “fear of falling,” “insufficient time,” and “low expectations regarding the effectiveness of exercise,” which significantly overlap with our findings. These barriers underscore the importance of therapists addressing patients’ perceptions and convictions about the advantages of exercise when developing tailored interventions^16^.

The clinical implications of our study are multifaceted. First, it is important to thoroughly assess each patient’s dyskinesia pattern before designing an exercise program. Second, it is essential to consider the individual’s exercise capacity by providing a wide range of options with varying levels of exercise complexity. Reducing external barriers to exercise is essential, as these factors play a key role in increasing participation and improving outcomes. A personalized rehabilitation approach that considers both exercise and psychosocial factors is likely to be most effective in improving patient compliance and quality of life.

This study provides factors that influence participation in exercise programs in patients with PD. One of the key findings of the study is that dyskinesia is not simply considered a symptom of PD, but rather an important determinant of participation in exercise activities. Of particular note is the fact that the impact of dyskinesia on exercise compliance is often underestimated in rehabilitation program design.

This study provides deeper insight into the needs and challenges faced by people with PD, paving the way for the creation of more impactful exercise plans that can significantly improve patients’ well-being while alleviating symptoms associated with PD.

### Limitations

Limitations should be acknowledged. First, the small sample limits the generalizability of the results, especially to subgroups with progressive movement disorders or limited participation. Future studies that include a larger population and subgroups of patients with severe dyskinesia may provide deeper insight into these complexities. In addition, the cross-sectional methodology of this study limits the ability to infer sustained effects over time. Longitudinal studies that monitor patients at extended intervals may provide more robust evidence regarding the consistency and sustained impact of exercise programs on overall health indicators.

In addition, while self-reported data have provided important insights into patient experiences, including objective measures such as step counts during “inactive” periods can increase the accuracy and reliability of the results. This approach can help us understand more precisely the variation in patients’ levels of physical activity and engagement and can better capture differences during periods of irregular exercise activity.

## Conclusion

These findings collectively highlight a complex relationship between Parkinson’s disease (PD) characteristics and participant engagement in moderate aerobic programs. While participants reported high satisfaction with approaches like TANGO and WALK, program compliance varied significantly, primarily due to motor complications, especially dyskinesia. In contrast, cognitive function, as assessed by MoCA scores, showed a strong correlation with higher satisfaction levels. Importantly, FOG did not significantly impact on program satisfaction or compliance, suggesting it may not hinder engagement in these moderate aerobic programs for PD. Ultimately, these findings underscore the critical need for tailored exercise programs that address both cognitive and motor symptoms to enhance adherence and maximize therapeutic outcomes for individuals with PD.

## Figures and Tables

**Figure 3:**
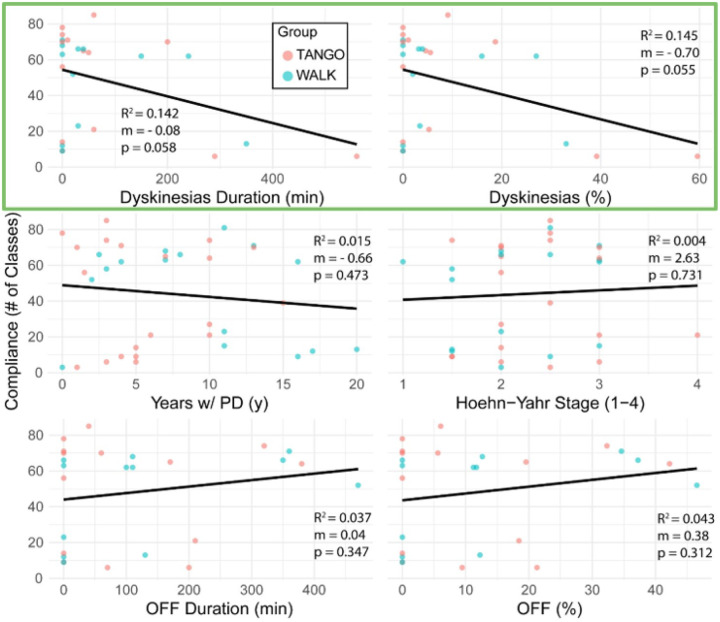
Participants and Exercise Program Compliance

**Figure 4:**
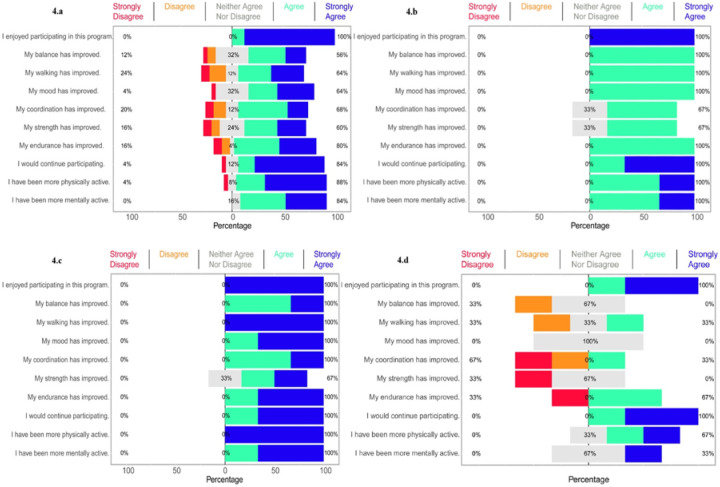
Participant Satisfaction Across Different Compliance and Dyskinesia Subgroups

**Figure 5:**
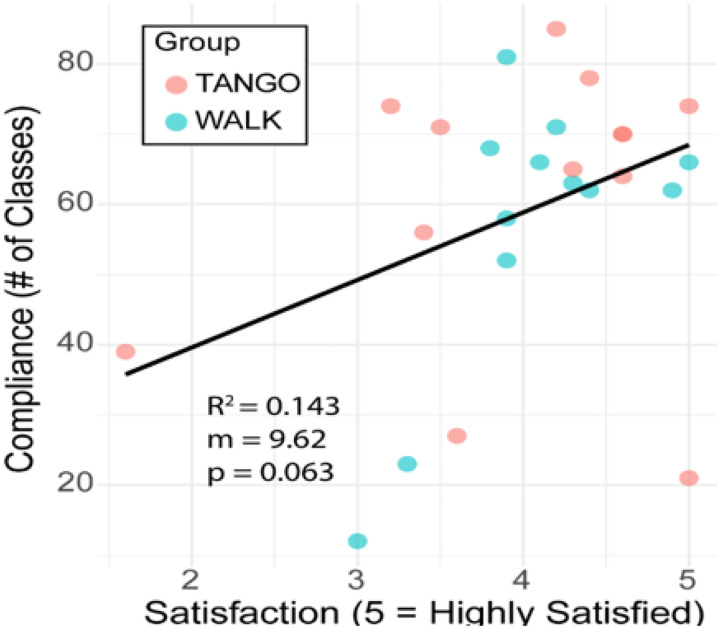
Satisfaction and Compliance within the total sample

**Table 1.** Demographic and clinical characteristics in groups (WALK, TANGO)

	TANGO (N=20)	WALK (N=16)	p
**Age (y)**	Mean (SD): 70.3 (7.7)	Mean (SD): 71.1 (7.7)	0.78
	Range: 49.0 – 83.0	Range: 57.0–82.0	
**Sex (F/M)**	Female: 6 (30%)	Female: 3 (19%)	0.44
	Male: 14 (70%)	Male: 13 (81%)	
**Race**	Asian: 1 (5%)	Asian: 0 (0%)	0.58
	Black/African American:5 (25%)	Black/African American: 4 (25%)	
	Hispanic or Latino: 1 (5%)	Multicultural: 1 (6%)	
	Other: 1 (5%)	White/Caucasian: 11 (69%)	
	White/Caucasian: 12 (60%)		

P<0.05 is significant

**Table 2.** Physical and PD-related clinical characteristics

**OFF (%)** ^**◊**^	Mean (SD): 12.4 (15.2)	Mean (SD): 15.4 (17.6)	0.69
	Range: 0.0–42.2	Range: 0.0–46.5	
**Off Duration** ^**◊**^	Mean (SD): 118.0 (143.7)	Mean (SD): 150.0 (177.1)	0.66
	Range:0.0–380.0	Range:0.0–470.0	
**Dyskinesia (%)** ^**◊**^	Mean (SD):4.4 (5.9)	Mean (SD): 5.5 (8.9)	0.75
	Range:0.0–18.7	Range: 0.0–27.0	
**Dyskin. Duration** ^**◊**^	Mean (SD): 42.0 (61.2)	Mean (SD): 51.0 (80.2)	0.78
	Range:0.0–200.0	Range:0.0–240.0	
**Hoehn-Yahr Stage**	Mean (SD):2.3 (0.5)	Mean (SD):2.1 (0.7)	0.35
	Range:1.5–3.0	Range:1.0–3.0	
**Years w/PD (y)**	Mean (SD):6.4 (4.8)	Mean (SD):8.5 (5.2)	0.32
	Range:0.0–15.0	Range:2.0–17.0	
**Compliance**	Mean (SD):61.1 (19.9)	Mean (SD):57.0 (19.9)	0.61
	Range:21.0–85.0	Range:12.0–81.0	
**Satisfaction**	Mean (SD):4.0 (0.9)	Mean (SD):4.1 (0.6)	0.85
	Range:1.6–5.0	Range:3.0–5.0	

## Data Availability

The data supporting this study’s findings are available from the corresponding author upon reasonable request.
